# Flux of the biogenic volatiles isoprene and dimethyl sulfide from an oligotrophic lake

**DOI:** 10.1038/s41598-017-18923-5

**Published:** 2018-01-12

**Authors:** Michael Steinke, Bettina Hodapp, Rameez Subhan, Thomas G. Bell, Dominik Martin-Creuzburg

**Affiliations:** 1 School of Biological Sciences, University of Essex, Wivenhoe Park, Colchester, CO4 3SQ United Kingdom; 2 University of Konstanz, Limnological Institute, Mainaustrasse 252, 78464 Konstanz, Germany; 3 Plymouth Marine Laboratory, Prospect Place, The Hoe, Plymouth, PL1 3DH United Kingdom

## Abstract

Biogenic volatile organic compounds (BVOCs) affect atmospheric chemistry, climate and regional air quality in terrestrial and marine atmospheres. Although isoprene is a major BVOC produced in vascular plants, and marine phototrophs release dimethyl sulfide (DMS), lakes have been widely ignored for their production. Here we demonstrate that oligotrophic Lake Constance, a model for north temperate deep lakes, emits both volatiles to the atmosphere. Depth profiles indicated that highest concentrations of isoprene and DMS were associated with the chlorophyll maximum, suggesting that their production is closely linked to phototrophic processes. Significant correlations of the concentration patterns with taxon-specific fluorescence data, and measurements from algal cultures confirmed the phototrophic production of isoprene and DMS. Diurnal fluctuations in lake isoprene suggested an unrecognised physiological role in environmental acclimation similar to the antioxidant function of isoprene that has been suggested for marine biota. Flux estimations demonstrated that lakes are a currently undocumented source of DMS and isoprene to the atmosphere. Lakes may be of increasing importance for their contribution of isoprene and DMS to the atmosphere in the arctic zone where lake area coverage is high but terrestrial sources of BVOCs are small.

## Introduction

Surface-to-atmosphere emissions of reactive BVOCs control the atmosphere’s oxidation capacity and secondary aerosol formation. These aerosols contribute considerably to the formation of particles affecting biogeochemical cycling, atmospheric processes, climate, and regional air quality in terrestrial^1^ and marine atmospheres^2^. Although lakes are recognised as hot-spots for CO_2_ exchange and the release of methane^3^, freshwater biomes have received little attention for their total contribution to the atmospheric BVOC burden. Here, we demonstrate a flux of isoprene (2-methyl-1,3-butadiene; C_5_H_8_) and DMS ((CH_3_)_2_S) out of Lake Constance and suggest that oligotrophic lakes can be a source of these BVOCs to the overlying atmosphere. Our findings are of particular importance for our understanding of BVOC emissions at night and suggest that lakes may sustain a substantial flux to the atmosphere at high latitudes where lake area density is exceptionally high but terrestrial emission very low.

Isoprene comprises about a third of all BVOCs in the terrestrial atmosphere and is recognised for its function in the physiological acclimation in vascular plants^4–6^. In contrast, this gas is unreported in lakes despite the demonstration that heterotrophic bacteria^7^, marine cyanobacteria, phytoplankton and seaweeds also produce isoprene^8^. Two biosynthesis pathways exist for isoprene that result in isopentenyl diphosphate, the universal isoprenoid precursor. They are named after their key intermediate metabolites, mevalonate (MVA) and 2-*C*-methyl-d-erythritol 4-phosphate (MEP). Under low light heterotrophic growth conditions, several freshwater eukaryotic microalgae and a cyanobacterium differentially expressed one or both pathways^9^ but the production of isoprene by freshwater biota is undocumented and not represented in Earth system models.

Marine environments are a predominant source of DMS^10^ and various physiological and ecological functions have been attributed to the production of this BVOC from its cellular precursor dimethylsulfoniopropionate (DMSP) in algae and bacteria^11^. These include cryoprotection, an overflow mechanism under unbalanced algal growth, as grazing deterrents, an antioxidant system that quenches reactive oxygen species^10^ or as chemical cues^12^. Molecular genetic evidence for various DMSP catabolic pathways that produce DMS exists for bacteria, fungi and algae^13,14^. DMS is also produced by trees and soils^15^ and in freshwater systems^16,17^. However, eutrophic lakes are suggested to be a minor source of DMS-sulfur to the atmosphere during periods of stratification since increased concentrations are associated with the anoxic hypolimnion^16^, likely as a result of microbial biomethylation of hydrogen sulfide^17^.

Concentrations and production rates of isoprene and DMS have previously been reported for estuarine and marine environments^18–23^ and such information has facilitated the estimation of the source strength of these climate-active BVOCs to the atmosphere^24,25^. A transect study from the North to South Atlantic^21^ indicated that isoprene and DMS do not correlate with concentrations of chlorophyll-*a* (chl-*a*) but positively correlate with the concentration of 19′-hexanoyloxyfucoxanthin, an accessory pigment occurring in the primarily marine haptophyte and some dinoflagellate algae, in areas characterised by low nitrogen concentrations. Limited information exists on the production and flux of DMS from lakes and freshwater sediments^26,27^ but similar data for isoprene is lacking. This shortage of ecosystem observations precludes the accurate estimation of global gas fluxes^15^.

BVOCs have important roles for the physiology of producers and consumers in aquatic food webs^12,28^. Isoprene and DMS are produced in response to oxidative stress from, for example, high light and temperature conditions in terrestrial plants (isoprene:^29^), phytoplankton (isoprene:^30^; DMS:^31^) and air exposure in corals (DMS:^32^). Further evidence suggests that the strong relationship between isoprene and photoprotective carotenoids in marine phytoplankton could relate to a photoprotective function^33^ and that marine phytoplankton use DMS and/or isoprene to mitigate ROS-induced metabolic damage under sublethal environmental stresses^6^. Hence, it is possible that the production of these BVOCs also assists with physiological acclimation to environmental conditions in freshwater phytoplankton. To date this has been largely unexplored.

This study investigated the concentrations of isoprene and DMS in Lake Constance (see Supplementary Fig. S1), the third largest body of freshwater in central Europe and a well-studied model for north temperate deep lakes. We quantified DMS and isoprene production in 10 species of freshwater algae from four different taxonomic classes using gas chromatography with flame-ionisation detection. Particular focus was on the vertical distribution of isoprene and DMS in depth profiles, their concentrations in surface samples over a diurnal cycle and the flux of these gases between Lake Constance and its overlying atmosphere.

## Results

### Depth Profiles

**Figure 1:**
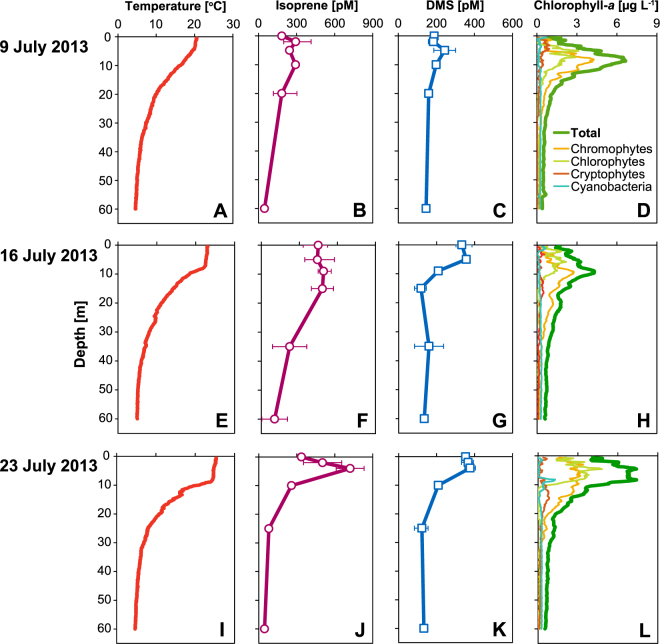
Depth profiles of temperature, isoprene, DMS and chl-*a* on 9, 16 and 23 July 2013. Concentrations of isoprene (**B**,**F**,**J**) and DMS (**C**,**G**,**K**) are shown as the arithmetic mean ± range of data (n = 2–3). Chl-*a* data (**D**,**H**,**L**) are shown as total and split based on fluorescence characteristics into four major phytoplankton groups (chromophytes, chlorophytes, cryptophytes, and cyanobacteria). Chl-*a* data were smoothed using a simple moving mean (running average) covering 0.80 ± 0.128 m depth.

Our weekly depth profiles showed a typical distribution of temperature and phytoplankton pigments in stratified lakes during summer with increasing stratification from 9–23 July 2013. We observed relatively high concentrations of isoprene (183 to 722 pM) and DMS (185 to 377 pM) associated with phototrophic processes in the epilimnion, which progressively deepened from approximately 4.5 to 8.3 m (Fig. 1). Lowest concentrations were generally found at the deepest sampling depth of 60 m (isoprene: 45 pM; DMS: 133 pM). Data from an optical profiler provided information on the vertical distribution of chl-*a* and fluorescence fingerprints were used to estimate the relative contribution of specific taxonomic groups to total chl-*a*. Total and taxon-specific chl-*a* (Fig. 1D,H,L) showed maxima at 8.7 m on 9 July (6.6 µg L^−1^), 9.0 m on 16 July (4.3 µg L^−1^) and 4.6 and 8.3 m on 23 July (both 7.4 µg L^−1^). The majority of biomass from the surface to the chl*-a* maxima had optical characteristics of chromophytes (including diatoms, dinoflagellates and chrysophytes: 36 to 43% of total chl-*a*) and chlorophytes (31 to 50%). Linear regression analysis indicated a significant positive correlation between BVOC and total chl*-a* concentrations (Pearson correlation, P ≤ 0.004, n = 18; for details see Supplementary Table S1). The taxon-specific data on chl*-a* concentration indicated significant positive correlations between isoprene and DMS with chlorophytes (Pearson correlation, P ≤ 0.003, n = 18), and between isoprene with chromophytes (Pearson correlation, P = 0.004, n = 18). Cryptophyte- and cyanobacteria-derived chl*-a* abundance was relatively low (2 to 17% of total chl-*a*) and did not correlate significantly with trace gas concentrations (P > 0.05). The fluorescence data provide a basic indication that the production of both BVOCs is relatively wide-spread across the different algal taxonomic groups.

### Phytoplankton incubations

The importance of phototrophic processes for the production of isoprene and DMS was confirmed by screening unialgal phytoplankton cultures of ten algal species from four algal classes. These measurements represent net rates resulting from the interplay between gross production and gross consumption processes in the alga and associated microbiota. After normalisation of our data to chl-*a* and carbon concentration in the phytoplankton cultures (Table 1), we found culture-specific production rates (Table 2) that ranged from no production of either isoprene or DMS (*Cyclotella meneghiniana*, *Chlamydomonas reinhardtii*, *Ulothrix fimbriata*) to isoprene only (*Cryptomonas* sp., *Anabaena variabilis*, *Microcystis aeruginosa*, *Synechococcus elongatus*), DMS only (*Chlorella vulgaris*, *Aphanizomenon flos-aquae*) or both isoprene and DMS production (*Scenedesmus obliquus*). This suggests that cryptophytes and cyanobacteria may have contributed to DMS and isoprene production in the lake despite the low abundance indicated by the optical profiler.

**Table 1:** Phytoplankton class, species and strain information, growth form, growth media, chlorophyll*-a* (chl-*a*) and particulate organic carbon (POC) concentrations in cultures used for trace gas production measurements. Algal cultures were grown in 4 L volumes at a temperature of 20 °C and a light intensity of ~100 µmol m^−2^ s^−1^ from fluorescent tubes. Cyanobacteria were grown in Cyano medium^74^, Chlorophyceae and *Cryptomonas* sp. were cultivated in Woods Hole (WC) medium either with or without vitamins^75^, and diatoms were grown in a modified M III medium with vitamins (M III KS)^76^. Data show mean ± standard deviation (n = 3).

Class and Species	Strain ID^a^	Growth form	Medium	chl-*a* [mg L^−1^]	POC [mg L^−1^]
Bacillariophyceae					
*Cyclotella meneghiniana*	SAG 1020-1a	Unicellular	M III KS + Vit	1.0 ± 0.21	71.4 ± 8.30
Chlorophyceae					
*Chlamydomonas reinhardtii*	SAG 11-31	Unicellular	WC	4.6 ± 0.62	73.2 ± 11.87
*Chlorella vulgaris*	SAG 211-11b	Unicellular	WC + Vit	10.1 ± 1.04	112.7 ± 3.17
*Scenedesmus obliquus*	SAG 276-3a	Unicellular	WC	5.8 ± 1.70	99.1 ± 17.48
*Ulothrix fimbriata*	SAG 36.86	Filamentous	WC	4.4 ± 0.42	94.9 ± 4.18
Cryptophyceae					
*Cryptomonas* sp.	SAG 26.80	Unicellular	WC + Vit	5.4 ± 0.43	106.1 ± 5.26
Cyanophyceae					
*Anabaena variabilis*	LI 81a	Filamentous	Cyano	6.4 ± 0.67	135.0 ± 16.63
*Aphanizomenon flos-aquae*	LI 83	Filamentous	Cyano	1.3 ± 0.02	42.1 ± 1.27
*Microcystis aeruginosa*	LI 78	Unicelluar	Cyano	1.4 ± 0.07	38.1 ± 0.63
*Synechococcus elongatus*	SAG 89.79	Unicellular	Cyano	4.6 ± 1.10	101.2 ± 24.59

^a^SAG = Culture collection of algae, University of Göttingen; LI = Culture collection of the Limnological Institute, University of Konstanz.

**Table 2:** Isoprene and DMS production in four classes of freshwater phytoplankton from 10 species after normalization to particulate organic carbon (POC) or chlorophyll-*a* (chl-*a*). ‘NS’ indicates that incubations with algae were not significantly different from controls with alga medium (two-tailed t-test, P > 0.05).

Class and Species	*n*	Isoprene		DMS	
		nmol [g org-C]^−1^ h^−1^	nmol [g chl-*a*]^−1^ h^−1^	nmol [g org-C]^−1^ h^−1^	nmol [g chl-*a*]^−1^ h^−1^
Bacillariophyceae					
*Cyclotella meneghiniana*	3	NS	NS	NS	NS
Chlorophyceae					
*Chlamydomonas reinhardtii*	3	NS	NS	NS	NS
*Chlorella vulgaris*	3	NS	NS	0.3 ± 0.03	3.5 ± 0.03
*Scenedesmus obliquus*	6	3.1 ± 2.31	49.2 ± 35.66	0.5 ± 0.28	9.0 ± 5.90
*Ulothrix fimbriata*	3	NS	NS	NS	NS
Cryptophyceae					
*Cryptomonas* sp.	3	0. 7 ± 0.53	12.6 ± 9.46	NS	NS
Cyanophyceae					
*Anabaena variabilis*	3	0.9 ± 0.15	18.7 ± 2.99	NS	NS
*Aphanizomenon flos-aquae*	3	NS	NS	0.7 ± 0.19	21.1 ± 5.30
*Microcystis aeruginosa*	3	6.2 ± 0.93	174.3 ± 27.21	NS	NS
*Synechococcus elongatus*	6	7.3 ± 1.63	159.3 ± 35.14	NS	NS

### Diel study

**Figure 2:**
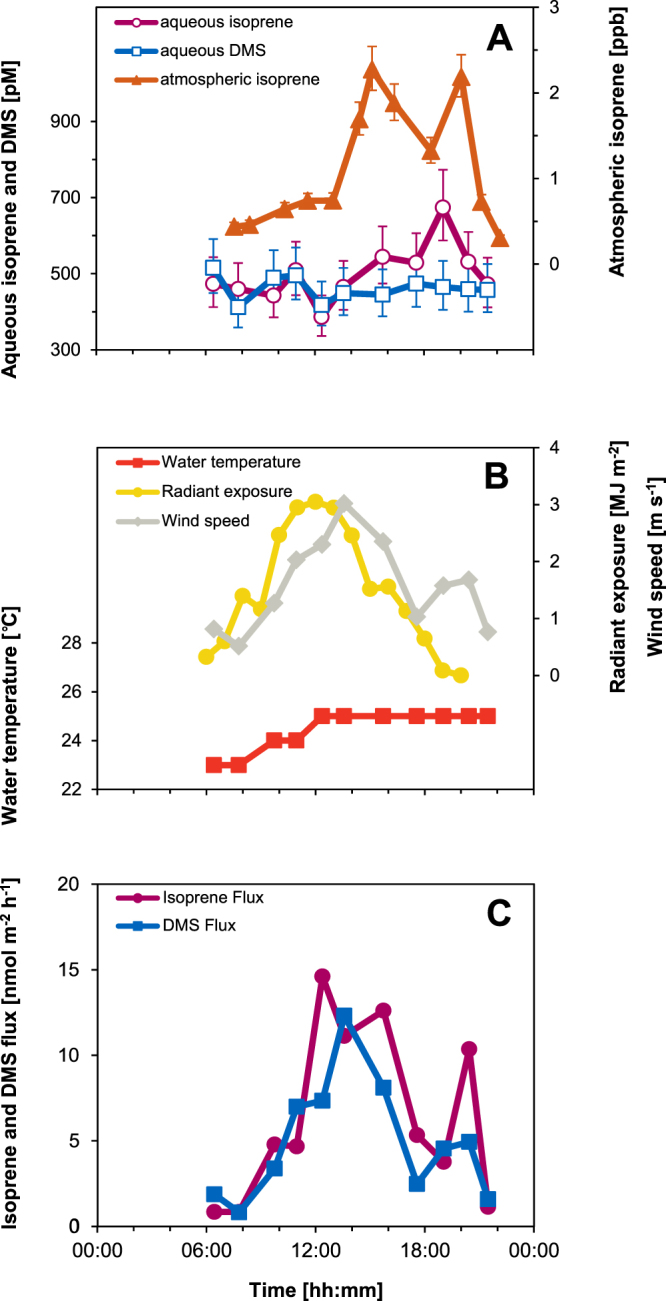
Diurnal study on 23 July 2013 showing concentrations of aqueous isoprene and DMS, and atmospheric isoprene (**A**), water temperature, radiant exposure and wind speed (**B**), calculated isoprene and DMS flux (**C**). Error bars in (**A**) indicate the coefficient of variation based on repeated calibrations.

We also explored the diurnal differences in lake isoprene and DMS since strong diel pattern of isoprene production are observed in seaweed incubations and rock pools^34^, and terrestrial environments have no or negligible isoprene in the atmosphere during the night^4,35^. Aqueous isoprene level was significantly lower in the morning than in the afternoon (Fig. 2A) with mean aqueous isoprene concentrations (±standard deviation) of 455 ± 44.7 pM between 06:25 h and 12:22 h, and 548 ± 75.7 pM between 13:34 h and 20:26 h (two-tailed t-test: P = 0.04, n = 5). The atmospheric concentrations of isoprene showed a similar pattern but concentration differences between morning and afternoon were more pronounced with a mean isoprene concentration of 0.6 ± 0.15 ppb between 07:34 h and 13:00 h and 1.9 ± 0.39 ppb between 14:27 h and 20:03 h (two-tailed t-test, P < 0.001, n = 5). No significant difference was observed in aqueous DMS between morning (466 ± 47.3 pM) and afternoon (459 ± 11.6 pM; two-tailed t-test, P > 0.05), with a mean concentration of 462 ± 31.0 pM (atmospheric DMS was below the limit of detection).

### Isoprene and DMS fluxes

Using air and water temperatures, and wind speeds (Fig. 2B), the concentration measurements allowed us to calculate the flux of isoprene and DMS across the water-atmosphere interface (Fig. 2C). Wind speeds were low throughout the diurnal study (1.6 ± 0.79 m s^−1^), constraining the transfer of gases into the atmosphere during our investigation. Isoprene flux was relatively small at the beginning (07:47 h: 0.8 nmol m^−2^ h^−1^) and towards the end of our diurnal study (21:29 h: 1.2 nmol m^−2^ h^−1^). Highest fluxes were observed between 12:22 h and 15:43 h (11.1 to 14.6 nmol m^−2^ h^−1^). Isoprene fluxes were likely similar at Sites 1 and 2 since they showed similar surface concentrations (around mid-day at Site 1: 337 pM on 23 July; Site 2: 387 pM on 25 July) and were driven by the diurnal variation in wind speed that directly affects the gas-transfer velocity used in our calculations. Using chl-*a* concentrations for the epilimnion on 23 July (48 mg m^−2^ or mean of 6.2 ± 1.36 µg chl-*a* L^−1^ from surface to 8.3 m depth), we can further calculate a biomass-normalised maximum isoprene flux of 304 nmol [g chl-*a*]^−1^ h^−1^. Flux of DMS showed a similar pattern to isoprene and ranged from 0.8 nmol m^−2^ h^−1^ at 07:47 h to a maximum of 12.3 nmol m^−2^ h^−1^ (256 nmol [g chl-*a*]^−1^ h^−1^) at 13:34 h to 1.6 nmol m^−2^ h^−1^ at 21:29 h.

## Discussion

We first compared isoprene concentrations and fluxes from the lake with measurements from a temperate mixed-deciduous forest of beech (48%), oak (44%) and birch (8%) at a location 416 km to the north-northeast of Lake Constance in July 2003. This is an example for a high isoprene-producing terrestrial environment in the northern European temperate zone where atmospheric isoprene concentrations ranged from near zero at night to about 3 ppb around noon indicating mean hourly fluxes from the terrestrial vegetation of 1 to 2 µg m^−2^ s^−1^ (equivalent to 53 to 106 µmol m^−2^ h^−1^)^35^. Using a conservative estimate of the leaf area index (5.5 m^2^ m^−2^ for beech and oak)^36^, this hourly flux equates to 10 to 19 µmol m^−2^ h^−1^ based on the one-sided leaf surface area. Our data on atmospheric isoprene concentrations were similar (0.3 to 2.3 ppb during the diurnal study) but flux from the lake (14.6 nmol m^−2^ h^−1^) was substantially lower than the flux from terrestrial vegetation. We also normalised the isoprene flux to depth-integrated chl-*a* for the lake epilimnion on 23 July, compared this with chl-*a* normalised terrestrial fluxes and find that these are about 80 to 160-fold higher than fluxes from the lake.

We then compared our measured fluxes with examples from low isoprene-producing terrestrial environments. The arctic tundra is relatively poorly vegetated and only 17–20% of plant species from cold environments produce isoprene^37^. Fluxes in Lake Constance are similar to typical fluxes from arctic tundra vegetation (9 to 39 nmol m^−2^ h^−1^)^38,39^. This raises the question whether arctic lakes may be a substantial source of isoprene to the local atmosphere, provided their production and resulting flux is similar to that of Lake Constance. Pelagic mean chl-*a* concentration in arctic lakes ranges from 0.3 to 5.6 µg chl-*a* L^−1^ (overall mean of 1.9 µg chl-*a* L^−1^)^40^ but the typically shallow arctic lakes have large parts of the benthic sediments located in the euphotic zone. This provides a surface for growth of attached algae resulting in substantial epilithic chl-*a* concentrations (258 to 458 mg m^−2^) that generate 28 to 77% of total primary production in six arctic lakes^41^. Although pelagic chl-*a* was higher in Lake Constance (6.2 ± 1.36 µg chl-*a* L^−1^), its morphometry suggests that epilithic primary production was small and restricted to the immediate shoreline. Furthermore, the taxonomic composition of arctic and subarctic lakes is similar to oligotrophic temperate lakes with frequent domination by diatoms and chlorophytes, cryptophytes only temporally important in the seasonal succession and a low abundance of cyanobacteria^42–45^. This generally matches the taxonomic composition of oligotrophic Lake Constance based on the fluorescence characteristics from the optical profiler that showed a high abundance of chromophytes (including diatoms) and chlorophytes, and a lower abundance of cryptophytes and cyanobacteria (Fig. 1). Taken together, this suggests that primary productivity of arctic lakes could likely support at least a similar isoprene flux as that of Lake Constance. Additionally, since lakes are an increasingly dominant feature in the landscape from northern temperate to arctic zones^46^ and much of the Arctic has an exceptionally high lake area density (limnicity) of 10 to 50%^47^, the relative importance of lakes in the release of isoprene to the atmosphere may exceed that of terrestrial sources in the arctic where lake area is large and terrestrial inputs are small. This suggests that, relative to terrestrial sources, lakes in cold-temperate and subarctic climates could add substantially to the local atmospheric isoprene burden.

We then assessed our data against measurements from marine environments. Using recent reviews on marine isoprene^33,48^, we calculated an overall mean marine concentration of 30 pM (mean range 4.7 to 126.7 pM; n = 12–14). Typical marine fluxes range from 2.8 nmol m^−2^ h^−1^ in the Southern North Sea^19^ to 313 nmol m^−2^ h^−1^ in the Southern Indian Ocean^49^. These fluxes are strongly controlled by wind speed owing to the relatively small isoprene concentrations in the marine atmosphere and its relatively high concentrations in seawater^34^. In comparison to the marine examples, isoprene concentration in Lake Constance was higher and ranged from 183 to 722 pM in the epilimnion. Even at the relatively low wind speed during our study, high concentrations resulted in a substantial flux (maximum of 14.6 nmol m^−2^ h^−1^ around noon). Hence, similar to the marine example, Lake Constance was an important source of isoprene to the local atmosphere with an extrapolated emission of 59 moles (4 kg) of isoprene on the day of our diurnal measurements alone. Since aqueous isoprene concentrations can build up during periods of low wind speed when loss due to water-to-air transfer is limited, Lake Constance also provides a reservoir of isoprene. It is further possible that, depending on wind conditions, isoprene flux can be sustained into the night-time as indicated by the increased flux from the lake when light intensity was relatively low and wind speed temporarily increased from 19:00 to 20:30 h (Fig. 2). We then simulated the potential flux of isoprene using night-time concentrations and temperatures from our diurnal study (see Supplementary Fig. S2) and wind-speed data for 28 July 2013 when the calm conditions during our measurements were interrupted by a three-hour moderate breeze (maximum of 6.8 m s^−1^), and calculated an initial flux of 49.0 nmol m^−2^ h^−1^. Hence, the lake likely acts as an important source of night-time isoprene when terrestrial production ceases due to the strong light and temperature dependency of biological isoprene production^4^. This night-time release is unrecognised but of particular relevance since day-time isoprene is predominantly and rapidly oxidised (lifetime of few hours) by the light-generated hydroxyl radical (•OH), whilst isoprene emitted during the night will be mostly rapidly oxidised by the typically 100-fold more abundant nitrate radical (NO_3_; formed from anthropogenic NO_2_ and ozone). This should then impact the type, yield and fate of the isoprene-nitrates formed locally and consequently the NO_X_ recycling, ozone and particle formation that may affect polluted urban atmospheres in the vicinity of lakes^50^.

DMS is the largest natural source of sulfur in the remote marine atmosphere and, similar to isoprene, may play some role in the formation and growth of atmospheric aerosol^51^ and impact on the night-time chemistry of the NO_3_ radical^50^. The transfer of DMS-sulfur into the atmosphere is estimated at 19.6 Tg per year^25^ which equates to a flux of 193 nmol m^−2^ h^−1^. As expected, the flux of DMS from Lake Constance (maximum of 12.3 nmol m^−2^ h^−1^) was lower than the marine flux and similar to the earlier estimates from Lake Kinneret that showed an estimated DMS-flux of 0.1 mmol m^−2^ month^−1^ (equivalent to 13.7 nmol m^−2^ h^−1^)^52^ and the mean flux from 10 Canadian lakes (7.1 nmol m^−2^ h^−1^) that, extrapolated to the Canadian boreal region, sustains an important 83% of biogenic sulfur in the atmosphere^27^.

Five of the phytoplankton cultures showed net-production rates for isoprene ranging from 12.6 to 174.3 nmol [g chl-*a*]^−1^ h^−1^ and three produced DMS at 3.5 to 21.1 nmol [g chl-*a*]^−1^ h^−1^. The isoprene production rates in the algal cultures were lower than the calculated lake flux after normalisation to chl-*a* biomass (304 nmol [g chl-*a*]^−1^ h^−1^). This could indicate that important isoprene-producing taxa were excluded from our screening or that environmental conditions (e.g. light, temperature) can significantly affect isoprene production rates in freshwater algae. This supports the idea that light-stress may drive the production of freshwater isoprene since it is linked to photoprotection in marine algae^6,33^. Our data agree with net-production rates in 21 marine algal strains from 7 taxonomic groups that varied by two orders of magnitude between strains (30 to 1340 nmol [g chl-*a*]^−1^ h^−1^)^8^. This suggests that the physiological processes involved in the production of isoprene are fundamentally similar between marine and freshwater environments.

As far as we are aware, surprisingly little information on the rates of DMS production in algal cultures is available in the literature. The high DMS-producing marine haptophyte *Emiliania huxleyi* (CCMP 373) produces DMS at rates of 10.1 ± 0.60 and 8.2 ± 1.80 nmol DMS L^−1^ h^−1^ during the day and night, respectively, at culture cell densities of 200 to 800 × 10^6^ cells L^−1^^53^. Using a cell density of 500 × 10^6^ cells L^−1^ and a mean chl-*a* concentration of 0.22 ng cell^−1^^54^, this equates to 91.6 ± 5.5 and 74.2 ± 16.4 nmol [g chl-a]^−1^ h^−1^. This is about 4 times higher than the DMS-production rate in our culture of the freshwater cyanobacterium *Aphanizomenon flos-aquae* but 21-times higher than in the chlorophyte *Chlorella vulgaris*. Marine dinoflagellates are among the highest producers of DMSP and DMS^55^. For example, the dinoflagellate symbiont *Symbiodinium* sp. produces DMS at 20 to 107 µmol [g chl-a]^−1^ h^−1^^56^, at least three orders of magnitude higher than the freshwater phytoplankton in our study.

It is likely that isoprene and DMS are of ecological importance^57–60^. Freshwater algae are recognised as a rich source of volatiles that are documented for their effects on drinking water quality^61^, and used as directional cues to find food in freshwater gastropods^62^, hence can affect food web structure and function^12^. It is timely and important to address the ecological and physiological relevance of isoprene and DMS in freshwater environments and assess their roles in the infochemistry and structuring of freshwater food webs.

## Methods

### Sampling sites

Water samples were collected in July 2013 from two sites in Upper Lake Constance, a large (571 km^2^), deep (z_max_ = 252 m), warm-monomictic, oligotrophic lake in south-western Germany at the northern fringe of the Alps (Supplementary Fig. S1). Site 1 was at the long-term sampling site of the Limnological Institute of the University of Konstanz located in Lake Überlingen, a fjordlike appendix of Upper Lake Constance, which was accessed via boat (47°45′43.6″N, 9°07′50.0″E; depth about 140 m). Site 2 was accessed via a mooring and located close to the Limnological Institute, about 30 m offshore (47°41′44.3″N, 9°11′38.1″E) with a water depth of about 3 m.

### Depth profiles

Water was collected from 6 depths (surface to 60 m) using a Niskin sampler at Site 1 at approximately 11:00 h on 9, 16 and 23 July 2013. Depths for discrete samples were selected based on *in situ* chl*-a* profiles recorded using a multi-channel fluorescence probe (bbe FluoroProbe, bbe Moldaenke, Schwentinental, Germany) and included samples from the surface (0 m) and from a maximum depth of 60 m. This probe has been shown to resolve the distribution of the four different taxonomic groups of chromophytes (including diatoms, dinoflagellates and chrysophytes), chlorophytes, cryptophytes, and cyanobacteria in laboratory cultures^63^ and lakes^64^ so that their abundances can be recorded based on fluorescence characteristics. For the quantification of discrete chlorophyll-*a* (chl-*a*) and organic carbon, samples were filtered immediately onto glass-fibre filters (Whatman GF/F; 25 mm diameter) and stored in a cool box before freezing filters at −20 °C at the Institute for subsequent analysis. For trace gas analysis, water was filled bubble-free into 250 mL gas-tight Winkler bottles (acid-washed and rinsed with ultrapure water prior to sampling) with a short length of silicone rubber tubing allowing for copious overflow before bottle closure. Samples were taken in analytical replicates (n = 3) and stored in a cool box equipped with several ice-packs before analysis of trace gases (n = 2 to 3) commenced ~1 hour after sampling.

### Diurnal study

Water was collected bubble-free using an inverted aspirator approximately every 1.5 h at Site 2 between 06:25 and 21:29 h on 25 July 2013. Water was transferred into gas-tight bottles as described above and analysis of trace gases commenced about 10 min later. Air samples were taken from outside the institute located in a rural setting approximately 80 m from the lake shore with an air intake at 7 m above the lake level by sucking air through a 10 m long 1/8 inch (3.2 mm) OD Teflon tube using a vacuum pump. Air was flushed for 10 min at 80 mL min^−1^ into the cryo-focussing apparatus to trap trace gases from the atmosphere as described below.

### Isoprene and DMS production in phytoplankton cultures

Algal cultures were aerated with compressed and filtered (0.2 µm pore size) air and grown under constant growth conditions using culture media depending on the cultures’ specific requirements (Table 1). The cultures were diluted by replacing 1 L of culture with fresh medium every 2 to 3 days and experiments were conducted 2 d after the last replacement.

On the day of the experiment, duplicate glass bottles were filled with algal medium (controls) or culture (treatment) at time zero (t_0_) and one bottle was immediately sacrificed for the quantification of isoprene and DMS. The other bottle was incubated under culture growth conditions and gases quantified at t_1_ after approximately 4 h. This was repeated twice using a staggered protocol resulting in 3 bottles each quantified for gases at t_0_ and t_1_. Treatments with significant difference to the controls (two-tailed t-test, P < 0.05) were considered for further analysis by subtracting control production rates and normalisation to culture chl-*a* and particulate organic carbon (POC) concentrations. It is important to note that previous incubation experiments with filtered seawater suggest that isoprene can also be produced at very low rates by photochemical processes with the bulk of this production controlled by ultraviolet light^65^. However, these experiments were affected by the presence of bacteria that could potentially lead to isoprene production from dissolved organic carbon. Furthermore, since we used borosilicate bottles and light derived from fluorescent tubes in our experiments photochemical production of isoprene was likely negligible during the incubations but small photochemical isoprene production may have added to the biological production processes at the lake surface.

### Quantification of discrete chl-*a* and POC

Glass-fibre filters (Whatman GF/F; 25 mm diameter) loaded with aliquots of the algal suspensions were used for photometric chl-*a* determination after wet extraction in ethanol^66^. Particulate organic carbon (POC) was quantified with an EuroEA3000 elemental analyser (HEKAtech GmbH; Wegberg, Germany; Table 1).

### Analysis of isoprene and DMS

Gas chromatography with flame ionisation detection combined with a purpose-built purge-and-trap system for the cryogenic enrichment of BVOCs was used for the analysis of isoprene and DMS following established protocols^8,67^ while using best practices for sampling and storage^68^. Calibration stocks for aqueous measurements of isoprene and DMS were volumetrically prepared, and a commercially-sourced isoprene gas standard was used for the calibration of atmospheric isoprene measurements. For method details see Supporting Information.

### Quantification of water-to-air flux

Concentrations of isoprene in water (*C*_*w*_) and air (*C*_*a*_) together with water temperature, air temperature and wind speeds measured at the Meteorological Station Konstanz (see Supplementary Fig. S1) were used to calculate water-to-air isoprene fluxes: *Flux* = *k*(*C*_*w*_ − *C*_*a*_ × *H*_*c*_), where *k* is the wind speed-dependent gas transfer velocity (cm hr^−1^)^69^, adjusted to the *in situ* Schmidt number^70^, and *H*_*c*_ is the Henry’s Law constant for isoprene (1.3 × 10^−2^ M atm^−1^)^71^. DMS flux calculations used the same approach and wind speed-based parametrisation of gas transfer velocity, but assumed *C*_*a*_ = 0 as atmospheric DMS levels were below the level of detection.

To compare the water-to-air flux with terrestrial flux estimates based on area or chl-*a*, we used a conservative estimate of the leaf area index of 5.5 m^2^ m^−2^ for beech and oak^36^, and literature data for chlorophyll (*a* + *b*) concentrations of 400 mg m^−2^ and chl-*a*/chl-*b* ratios in oak of 3.4^72,73^.

### Data analysis

Commercial software (GC Solution Lite version 2.41; Shimadzu UK, Milton Keynes, UK) was used for peak integration and data retrieval. We confirmed that test assumptions were met before conducting statistical analyses (two-tailed t-test and regression analysis) in MS Excel version 14.

### Data availability

The datasets generated and analysed during the current study are available from the corresponding author on reasonable request.

## Electronic supplementary material

Supplementary Information
