# Supplementary material for: Flux of the biogenic volatiles isoprene and dimethyl sulfide from an oligotrophic lake
Source: Sci Rep. 2018 Jan 12;8:630. doi: 10.1038/s41598-017-18923-5 (PMC5766545; doi:10.1038/s41598-017-18923-5)
Supplement: Supplementary file 1 — Supplementary Information [file 41598_2017_18923_MOESM1_ESM.pdf]

Supplementary Information

Flux of the biogenic volatiles isoprene and dimethyl sulfide from an oligotrophic lake

Authors

Michael Steinke[1,*], Bettina Hodapp[2], Rameez Subhan[1], Thomas G. Bell[3], Dominik Martin-Creuzburg[2]

Affiliations:

[1] School of Biological Sciences, University of Essex, Wivenhoe Park, Colchester CO4 3SQ, United Kingdom

[2] University of Konstanz, Limnological Institute, Mainaustrasse 252, 78464 Konstanz, Germany

[3] Plymouth Marine Laboratory, Prospect Place, The Hoe, Plymouth, PL1 3DH, United Kingdom

[*]*Corresponding author*

Michael Steinke

msteinke@essex.ac.uk

CONTENTS **Page number**

Supplementary Table **2**

Supplementary Figures **3 - 4**

Supplementary Methods **5 - 7**

Supplementary References **8**

Supplementary Table

Table S1. Linear regressions of taxon-specific and total chlorophyll with concentrations of isoprene and DMS for the depth profiles on 9, 16 and 23 July 2013. The slope, intercept, linear regression coefficient (r) and level of significance (P) are shown. Sample size (n) = 18; NS = not significant (P>0.05), significant regressions indicated in bold.

Chlorophyll	Isoprene				DMS			
	slope [pmol μg^{-1}]	intercept [pM]	r	P	slope [pmol μg^{-1}]	intercept [pM]	r	P
Chromophytes	109.9	159.5	0.650	**0.004**	36.0	176.2	0.427	NS
Chlorophytes	104.7	187.7	0.666	**0.003**	61.8	156.2	0.788	**<0.001**
Cryptophytes	4.9	297.8	0.006	NS	-15.4	225.5	-0.039	NS
Cyanobacteria	-468.9	412.2	-0.428	NS	-223.1	275.7	-0.408	NS
Total	63.6	120.1	0.699	**0.001**	29.3	139.4	0.647	**0.004**

Supplementary Figures

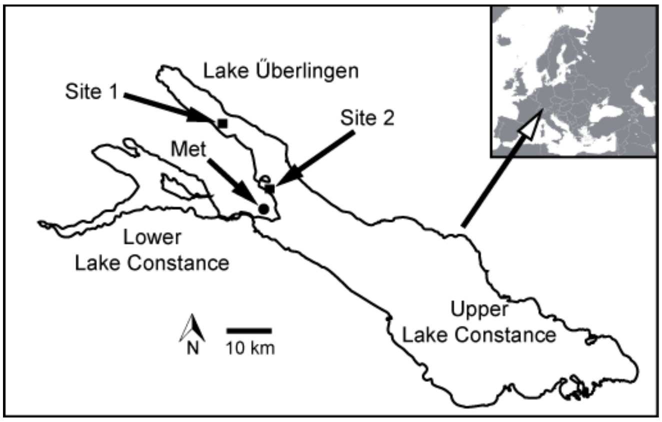

Fig. S1. Outline map of Lake Constance showing the location of the two sampling sites (Sites 1 and 2, indicated by black squares) and the position of the Meteorological Station Konstanz (Met, indicated by black circle). Inset shows map of Europe with open arrow indicating the location of Lake Constance. The maps were created from images obtained at https://commons.wikimedia.org/wiki/File:Blank_Template_for_Greater_Europe.PNG and https://commons.wikimedia.org/wiki/File:Bodensee_satellit.jpg using Adobe Illustrator CS version 11.0.0.

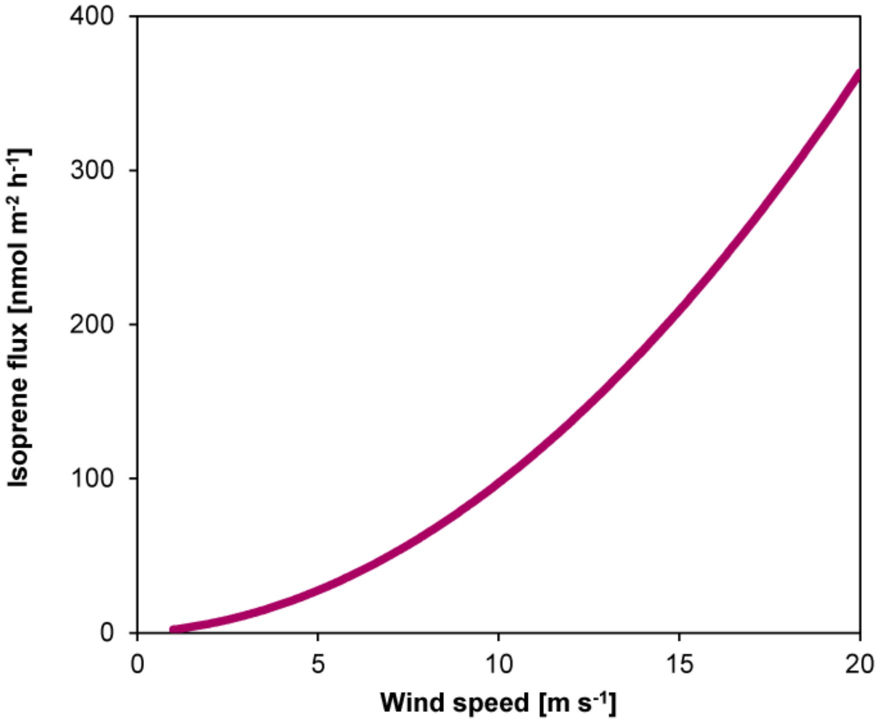

Fig. S2. Wind-dependent simulated potential flux of isoprene using night-time concentrations and temperatures from the diurnal study on 23 July 2013.

Supplementary Methods

Analysis of isoprene and DMS. A gas chromatograph equipped with a capillary column (50 m × 0.53 mm × 10 μm Rt-Alumina BOND/KCl; Restek, Saunderton, United Kingdom) and a flame-ionization detector (GC-FID model 2014; Shimadzu, Milton Keynes, United Kingdom) was used. The oven temperature programme was 2 min at 80 °C, ramp to 170 °C at 10 °C min^{-1}, followed by a ramp to 200 °C at 70 °C min^{-1} and 2 min held at 200 °C. Injector and detector were operated isothermally at 200 and 250 °C, respectively. Helium carrier gas was supplied at 15.56 ml min^{-1} (linear velocity 80 cm s^{-1}).

A purpose-built stainless-steel purge-and-trap apparatus for the cryogenic enrichment of trace gases was used (Exton et al 2013; Franchini and Steinke 2017). Using bubble-free sampling, 208 mL of sample was transferred from a Winkler bottle into the purge tube with a glass syringe equipped with a filter-holder and glass-fibre filter (Whatman GF/F, 47 mm diameter) before purging the filtrate with N_2 for 20 min at 80 mL min^{-1}. The sample gas was dried in two steps by first passing the sample stream through a condenser at 0 °C (emptied of condensate daily), before drying with a Nafion counter-flow drier (Permapure MD-050-72S-1, Fluid Controls Ltd., Aldermaston, UK) supplied with a counter-flow of dry N_2 at 240 mL min^{-1}. The sample gas was then passed into a stainless steel cryotrap (1/16 inch or about 1.59 mm OD) kept at a temperature of -160 °C using liquid N_2 and a purpose-built temperature controller. After the 20 min purge, the cryotrap was connected in-line with the GC carrier flow using a 6-port 2-position valve (C6UWE; VICI Valco International, Schenkon, Switzerland) and heated to 90 °C using freshly boiled water to transfer the enriched trace gases onto the GC column for separation and quantification. Under these conditions, the mean retention times (± standard deviation) of isoprene and DMS were 9.49 ± 0.067 min (n = 290) and 11.65±0.088 min (n = 62), respectively. Occasional blank measurements indicated that the system was free of hysteresis effects for isoprene and DMS.

Quantification of isoprene and DMS. Isoprene calibration stock for aqueous measurements was freshly prepared volumetrically. A small glass vial was placed into a gas-tight crimp-seal vial (20 mL nominal volume, Chromacol; Fisher Scientific, Loughborough, UK) to aid with mixing. The vial was then completely filled with 20.9078 mL ultrapure water and gas-tightly closed with a crimp-seal. For the primary stock, 10 µL of isoprene (solubility in water of 642 mg/l at 25 °C) was injected by gas-tight syringe into the sealed vial before shaking the vial to aid in complete dissolution of the isoprene (final concentration 4.78 mM). A secondary stock was prepared by diluting 10 µL of primary stock into a crimp-sealed vial filled with ultrapure water (final concentration 2.29 µM). Ten different volumes of this secondary stock (10 to 250 µL) were transferred via gas-tight syringe into the purge tube filled with 208 mL of ultrapure water resulting in concentrations of 110 to 2749 pM for the calibration of aqueous isoprene that yielded a linear regression coefficient (r^2) of > 0.99. For aqueous isoprene, we determined a level of detection (LOD; signal-to-noise ratio of 3:1) and level of quantification (LOQ; signal-to-noise ratio of 10:1) of 1.3 and 4.2 pM, respectively.

A similar procedure using 5 µL DMS to prepare the primary stock (final concentration 3.26 mM) and 5 µL of primary stock to prepare the secondary stock (final concentration 779 nM) was used for the DMS calibration. Eight different volumes (20 to 800 µL) were introduced into the purge tube filled with 208 mL of ultrapure water resulting in concentrations of 75 to 2995 pM for the calibration of aqueous DMS that yielded a linear regression coefficient (r^2) of > 0.98. For aqueous DMS, we determined an LOD and LOQ of 6.6 and 22.1 pM, respectively.

A commercial calibration gas (100 ppm isoprene in helium; Scientific and Technical Gases, Newcastle-under-Lyme, UK) was used for the gaseous isoprene calibration. Triplicate direct injections of five different volumes of calibration gas ranging from 10 to 40 µL (equivalent to 41 to 165 pmol) yielded a linear regression coefficient (r^2) of > 0.99. For gaseous isoprene, we determined an LOD and LOQ of 0.04 and 0.14 ppb, respectively. Calibration of gaseous DMS was not required since atmospheric concentrations were below the limit of detection.

Although our analytical system was built from stainless steel and not optimised for the quantification of DMS, we obtained a linear response in our calibrations without hysteresis effects suggesting that useful data were collected for isoprene and DMS.

Supplementary References

Exton DA, Suggett DJ, McGenity TJ, & Steinke M (2013) Chlorophyll-normalized isoprene production in laboratory cultures of marine microalgae and implications for global models. *Limnol Oceanogr* 58(4):1301-1311.

Franchini F, & Steinke M (2017) Protocols for the quantification of dimethyl sulfide (DMS) and other volatile organic compounds in aquatic environments. *Hydrocarbon and Lipid Microbiology Protocols*, eds McGenity TJ, Timmis KN, & Nogales B (Springer, Berlin), pp 161-177.
